# Predictors and prognosticators for survival with Yttrium-90 radioembolization therapy for unresectable colorectal cancer liver metastasis

**DOI:** 10.18632/oncotarget.16007

**Published:** 2017-03-08

**Authors:** Meaghan S. Dendy, Johannes M. Ludwig, Hyun S. Kim

**Affiliations:** ^1^ Drexel University College of Medicine, Philadelphia, PA, USA; ^2^ Department of Radiology and Biomedical Imaging, Division of Interventional Radiology, Yale School of Medicine, New Haven, CT, USA; ^3^ Yale Cancer Center, Yale University, New Haven, CT, USA

**Keywords:** colorectal cancer, liver cancer, radioembolization, Y^90^, biomarkers

## Abstract

This critical review aims to explore predictive and prognostic biomarkers of Yttrium-90 (Y^90^) radioembolization therapy of colorectal liver metastases. A brief overview of established predictive and prognostic molecular and genetic biomarkers in colorectal cancer therapies will be discussed. A review of the literature on imaging modalities, genetic, metabolic and other molecular markers and the subsequent outcomes in post-Y^90^ treatment will be presented. How these biomarkers and future biomarker research can inform locoregional treatment decisions in the clinical setting of metastatic colorectal cancer lesions of the liver will be explored. There are opportunities for personalized cancer treatment in the setting of Y^90^ radioembolization. The ability to predict tumor response after Ytrium-90 radioembolization therapy can greatly impact clinical decision making and enhance treatment outcomes, therefore further research into the field is needed.

## INTRODUCTION

Colorectal cancer (CRC) is the second most common cause of cancer-related death in both men and women [1]. At time of diagnosis around 20% of patients present with distant metastases, while up to 60% will develop distant metastases during the course of disease [2, 3]. The prognosis of patients with metastatic CRC (mCRC) is estimated to be only 11.7% within the first 5 years of diagnosis[1]. The liver is the most common site of metastases and liver involvement is thought to cause the majority of deaths in patients with metastatic CRC [4, 5].

### The role of radioembolization in the treatment of CRLM

Surgical liver resection is the standard treatment for CRC metastases of the liver. However, only an estimated 20-30% are deemed curatively resectable [6, 7]. Neoadjuvant chemotherapy has been shown to downstage 10-30% of patients to allow for subsequent resection [8, 9]. Fluropyrimidine 5-fluorouracil has been in use for the past few decades while newer agents like oxaliplatin, irinotecan and inhibitors of VEGF and EGFR have been increasingly used with improved median survivals of more than 2 years [10–12] . There remains a vital need for treatment options in those cases that are considered unresectable and are also refractory to chemotherapy. Locoregional therapies have been developed in the past two decades and are evolving as primary treatment options in the process of downstaging disease to allow for curative resection. Y-90 radioembolization has shown promising results as a tool in the management of unresectable CRC liver metastases.

Y-90 radioembolization has been recommended for chemorefractory CRC patients who have primarily liver metastases and in those patients who wish to avoid systemic chemotherapy treatment [13]. Ideal candidates for radioembolization should be at least 18 years old with ECOG scores of ≤2, serum bilirubin <3.0mg/dL, granulocyte count >1.5×10^9^, serum creatinine <2.0 mg/dl, platelet count >50×10^9^, and have adequate pulmonary function [14, 15]. There have been some encouraging studies that have shown good response in salvage patients. One study by Kennedy et al. demonstrated median OS of 10.5 months in treatment responders, compared to that of non-responders (4.5 months (*p* = 0.0001)) [16]. Hendlisz et al. conducted a prospective, randomized phase III trial showing that radioembolization combined with chemotherapy lengthens the time-to-progression in CRLM after having progressed on initial systemic treatment. Time to progression was 4.5 months when combined with Y-90 treatment, as compared to 2.1 months in the cohort that only received chemotherapy (*p* = 0.03) [17]. Despite the many studies supporting its safety and low rate of toxicity, the NCCN guidelines for treatment of metastatic colorectal cancer lacks consensus amongst panel members and it currently remains a Category 3 treatment [18]. Further prospective randomized clinical trials are required before Y-90 radioembolization can be definitely established as primary treatment option.

**Table 1a:** Imaging Predictors

Study	Study Design	Number of Subjects	Outcome
Jiao et al. and Szyszko et al. (2007)	retrospective	10 patients	PET better than CT at assessing response
Flamen et al. (2008)	prospective	8 patients (39 liver lesions)	cut-off value of 1 for the MAA-tumor-to-normal uptake ratio estimates a favorable outcome
Dudeck et al. (2010)	prospective	21 patients (41 lesions)	ADC decreased significantly in responding lesions
Tochetto et al. (2010)	retrospective	28 patients (74 lesions)	decreased attenuation on CT correlates with PET-FDG uptake and anticipates treatment success
Gulec et al. (2010)	prospective	20 patients	favorable outcomes: pre-treatment FTV <200cc, TLG <600 g; post-treatment FTV >30cc, TLG of <100 g
Zerizer et al. (2012)	retrospective	25 patients (121 lesions)	PET/CT correlates with the responses of biomarkers (LDH, CEA, CA 19-9)
Fendler et al. (2013)	prospective	80 patients	responders with change in TLG had longer survival
Lam et al. (2013)	retrospective	25 patients	SPECT-based calculation of DT correlated with radiographic response, decrease in serum CEA, and OS
Soydal et al. (2013)	prospective	35 patients	ΔTLG >26.5 showed increased survival
Ulrich et al. (2013)	retrospective	66 patients (435 lesions)	no association of response with (99m)Tc-MAA uptake or with catheter position
Kennedy et al. (2015)	retrospective	195 patients	RECIST 1.0 responders showed increased survival
Sabet et al. (2015)	retrospective	51 patients	increased OS via PET response; Hepatic tumor burden >25 % showed decreased survival
Fowler et al. (2015)	prospective	9 patients	increased dose (avg of 29.8 Gy) anticipated response
Barabasch et al. (2015)*	prospective	20 patients	sensitivity for detecting response was significantly higher for MRI than for PET
Chapiro et al. (2015)	retrospective	29 patients	EASL and mRECIST did not predict patient survival; qEASL was sole predictor of patient survival
Schmeel et al. (2016)	retrospective	44 patients	ADC changes on DWI can predict survival

*- study was conducted on mCRC patients as well as other liver malignancies

Selection of optimal patients for Y-90 treatment is key, as it is vital in interventional oncology to allow for the best possible patients outcomes and also to lower health care costs by avoiding unbeneficial therapies. Patient selection parameters currently in use have not been optimized and have lead to possible over- and under-treatment using these therapies. As such, there is an opportunity in this area to enhance patient selection and patient outcomes by identifying biomarkers that will better guide treatment decisions.

### The role of biomarkers for the treatment of mCRC with ^90^Y-radioembolization

The National Institutes of Health define Biomarkers as “a characteristic that is objectively measured and evaluated as an indicator of normal biological processes, pathogenic processes, or pharmacologic responses to a therapeutic intervention”[19].

Research has shown that a number of biomarkers can predict survival outcomes in patients with metastatic CRC. Genetic markers as well as metabolic and imaging markers have been shown to be predictive of tumor growth and metastatic spread. In a clinical setting the use of these markers can lead to important treatment decisions in regards to which lesions might be more susceptible to specific treatments to improve patient care. The purpose of this review is to summarize and discuss the current literature behind biomarkers of CRC, including metabolic, genetic, tumor and imaging markers that have been tested and evaluated for Y-90 radioembolization to better inform interventional oncologist on treatment decisions and spark interest in this research field.

**Table 1b:** Molecular and Genetic Predictors

Study	Study Design	Number of Subjects	Outcome
Gray et al. (1989)	retrospective	10 patients	patients who received >30 Gy had at lease a 50% decrease in serum CEA
Boppudi et al. (2006)	retrospective	54 patients	CEA levels decrease faster than decrease in lesion size via CT
Fahmueller et al. (2012)	prospective	49 patients	increased CEA, CA 19-9, CYFRA 21-1, CRP, LDH, AST, CHE, GGT, alk phos, amylase and nucleosomes suggest poor outcomes after Y-90
Fahmueller et al. (2013	prospective	49 patients	high HMGB1 were associated with poor outcome
Melucci et al. (2013)	prospective	50 patients	reduction of survivin, p53, and Bcl-2 expression post-Y90 suggest a favorable outcome
Carpizo et al. (2014)	prospective	22 patients	poor outcomes: increased baseline Ang-2 and IL-8; transient increases in VEGF and PDGF-BB post-Y-90
Tohme et al. (2015)	retrospective	104 patients	decreased survival of patients with high NLR
Henrie et al. (2015)	retrospective	12 patients	increased albumin associated with increased OS
Lahti et al. (2015)	retrospective	104 patients	KRAS wt patients have increased survival advantage
Magnetta et al. (2016)	retrospective	82 patients	PFS was longer in KRAS wt patients

### Predictive biomarkers of Ytrium-90 (Y90) radioembolization therapy in metastatic CRC

#### Molecular and genetic biomarkers

Limited information exists on biomarkers of CRC patients with liver metastases who undergo radioembolization treatment. The first molecular biomarker study completed in this population was a retrospective study by Gray et al. in 1989 (*n* = 10). The group explained that patients who received >30 Gy had at least a 50% decrease in serum carcinoembryonic antigen (CEA)[20]. Though this was only completed in a small 10 patient cohort and the study was done via laparoscopic surgery, it was an early demonstration as to how biomarkers can guide our understanding of treatment success. More recently, Boppudi et al. (*n* = 54) found that CEA levels fell rapidly (>75%) within 2 months of selective internal radiation therapy (SIRT) and found it to be more reliable in determining treatment response than CT scanning completed soon after treatment of mCRC liver lesions. The data showed that it took 3-21 months (median 12 months) for tumor size to maximally decrease post-treatment [21]. The group theorized that CEA levels are more representative of tumor response than imaging is due to the destroyed microvasculature of the tumor post-radioembolization treatment. This destruction leads to decreased efficiency of macrophage response and resulting cleanup. Another study, from Tohme et al. (*n* = 104), has shown via univariate and multivariate analysis that elevated neutrophil-lymphocyte ratio (NLR) in preoperative patients receiving radioembolization for CRC liver metastases is associated with poor survival outcomes (median OS high NLR = 5.6 mos; low NLR = 10.6 mos (*p* = 0.001)) [22]. The same group was able to show that patients with low neutrophil-lymphocyte ratio treated with radioembolization attained a survival advantage, which has been corroborated by other reports [23, 24]. The science behind this is not yet fully understood, but is thought to be caused by the decreased lymphocytic response to tumor cells and the increased angiogenic factors released by neutrophils. Both these factors would give the tumor cells a survival advantage and would decrease the efficacy of radioembolization treatment. Fahmueller et al. (*n* = 49) completed a prospective study that showed CEA, CA 19-9, CYFRA 21-1, CRP, LDH, AST, choline esterase (CHE), gamma-glutamyl-transferase (GGT), alkaline phosphatase, and amylase (all 0 h, 24 h) and nucleosomes (24 h) were found to be prognostic relevant markers (*p* < .05) of survival in univariate analysis. In Cox-regression multivariate analysis the combination of CRP with AST was found to show the most significance with regards to survival implications. It should also be noted that these studies were all completed with blood samples as opposed to tumor biopsies [25]. Another study from the same group (*n* = 49) showed that high mobility group box 1 protein (HMGB1) levels at 24 hours post-treatment were higher in patients with disease progression as compared to those without progression of disease. Overall survival statistics showed that high pretherapeutic (0 hr) and 24 hr levels of HMGB1 were associated with poor outcomes (at 0hr: median OS 19.6, 15.4, 7.8, 4.6 in Quartiles 1-4, *p* = .012; at 24 hr: median OS 6.8, 13.8, 4.8 in Quartiles 2-4, *p* = .004) [26]. A prospective study completed by Melucci et al. (*n* = 50) with biopsy samples found that survivin (92.3% vs 53.8%; *p* = 0.06), p53 (100% vs 69.2%; *p* = 0.05) and Bcl-2 (69.2% vs 53.8%; *p* = 0.05) expression decreases after radioembolization treatment and may inform radiosensitivity of CRC liver metastases [27]. The group postulated that the change in these biomarkers post-Y-90 treatment might be due to epigenetic changes or clonal selection. Another small prospective study was completed by Carpizo et al. (*n* = 22) and showed Ang-2 (*p* = .033) and IL-8 (*p* = .041) both had higher baseline levels in patients with decreased overall survival (OS) (<6 months). Patients with OS ≤6 months were found to have transient increased levels of VEGF and PDGF-BB post-Y90 therapy compared to patients with OS > 6 months after treatment indicating their possible use as prognostic biomarkers post-treatment [28]. These findings support the concept that radioembolization treatment may enhance angiogenesis signaling and the possible utility in pretreating patients receiving Y-90 therapy with antiangiogenic therapies. Henrie et al. (*n* = 12) then showed increased albumin was found to be significantly associated with increased OS for mCRC after radioembolization treatment (*p* = 0.02, HR = 0.02, 95% CI: 0.001–0.52.) [29].

**Table 1c:** Tumor and Patient Characteristics

Study	Study Design	Number of Subjects	Outcome
Dunfee et al. (2010)*	prospective	130 patients*	ECOG performance status >0, hepatic tumor burden of 51%-75%, bilirubin level >1.3 mg/dL, response based on WHO criteria, and lymphocyte depression yield worse outcomes
Deipolyi et al. (2014)	retrospective	62 patients	higher LSF had significantly decreased survival; pts who received chemotherapy before Y90 had low LSF had the longest survival
Schonewolf et al. (2014)	retrospective	30 patients	tumor volumes <300 mL were predictive for extrahepatic failure patterns
Tohme et al. (2014); HPB	retrospective	107 patients	no significant difference was found with regard to age, presence of extrahepatic disease at time of Y90 was associated worse survival
Sofocleous et al. (2015)	prospective	53 patients	CEA levels ≥ 90 ng/mL and microscopic lymphovascular invasion of the primary were predictors of decreased OS
Abbott et al. (2015)	retrospective	68 patients	OS for patients with ≤ 25% HBD was better
Fendler et al. (2015)	retrospective	100 patients	reduced patient survival: no liver surgery before Y90), CEA serum level ≥150 ng/ml, transaminase toxicity level ≥2.5x upper limit of normal, and summed CT size of the largest two liver lesions ≥10 cm
Xing et al. (2016)	retrospective	79 patients	high LSF demonstrated poorer survival compared with low LSF

*- study was conducted on mCRC patients as well as other liver malignancies

The first genetic mutation study in this patient population was completed by Lahti et al. (*n* = 104), who showed that unresectable CRC liver metastases with wild type KRAS show greater response to Y-90 radioembolization than those lesions with mutant KRAS. Lahti et al. showed that median OS from first Y-90 radioembolization was significantly greater in KRAS wt patients (9.5 mo vs 4.8 mo; *p* = .041) [30]. This was corroborated in a recent study by Magnetta et al. (*n* = 82), which showed PFS was longer in KRAS wt (median 166 days [95% CI 96-258 days]) vs. mut (median 91 days [95% CI 79-104 days], *p* = 0.002). The study also showed that KRAS mut patients were 1.48 times more likely to progress at first follow-up imaging than wt (95% CI 1.06–2.08, *p* = 0.024) [31]. This correlation may be due to the ability of KRAS mut tumors to metastasize more readily or also could be due to resistance to radiation-induced apoptosis present in these tumor cells [30].

#### Imaging markers

Other forms of predictors are needed to form better therapeutic strategies where radioembolization of CRC liver metastases is concerned. There is ample research available in support of imaging studies being used soon after Y-90 radioembolization to inform a more accurate assessment of tumor response. Jiao et al. and Szyszko et al. (*n* = 10) reported on the use of PET scan response being more successful in measuring tumor response than CT scans. The data showed that the PET scan mean pre-treatment SUV was 12.2+/-3.7 as compared to the post-treatment SUV of 9.3+/-3.7. Only 13% of the same tumors had reduced in size on CT after Y-90 treatment [32, 33]. The presence of necrosis, hemorrhage and cystic changes on CT makes measurements difficult in the immediate post-treatment setting, whereas the change in SUV values can be seen earlier and assessed more accurately [34]. Tochetto et al. (*n* = 28) then revealed that post-treatment attenuation correlates with metabolic activity on PET-FDG and can be used to anticipate treatment success. The lesions treated in this study had decreased diameter, volume, and attenuation posttreatment via CT (*p* < .05). Percent change in attenuation had higher correlation with percent change in SUV (*r* = 0.61) than diameter (*r* = 0.39) or volume (*r* = 0.49) when compared, and that ≥15% decreased attenuation showed 84.2% sensitivity and 83.3% specificity in predicting response at FDG-PET evaluation [35]. Zerizer et al. (*n* = 25) used a retrospective model to look at the superiority of F-FDG PET/CT and its correlation with the responses of tumor markers after Y-90 treatment. The study results showed that PET/CT is superior in response assessment to RECIST or tumor density measurements [36]. Fendler et al. (*n* = 80) also found that using RECIST criteria did not predict survival but also correlated the PET response to survival. The responders with change in metabolic volume or total lesion glyscolysis (TLG) were shown to survive longer (92 vs. 49 wk (*p* = 0.006) and 91 vs. 48 wk (*p* = 0.025)) [37]. Another study by Sabet et al. (*n* = 51) supported this by showing early metabolic responders survived longer than non-responders (*p* < 0.001) with a median OS of 10 months (95 % CI 3-16) versus 4 months (95 % CI 2-6). Sabet et al. also divulged that hepatic tumor burden impacted treatment outcome (*p* < 0.001) with a median OS of 5 months (95 % CI, 3-7) for patients >25 % metastatic liver replacement compared to 14 months (95 % CI 6-22) for the less advanced patients [38]. The study by Soydal et al. (*n* = 35) again corroborates the association between PET response and survival showing that a change in TLG >26.5 yielded longer survival (20.76 +/- 2.71 (95% CI 15.46-26.06) mos) compared to a change in TLG <26.5 (11.32 +/- 1.18 (95% CI 9.02-13.62 mos)(*p* = 0.016) [39]. A large retrospective study by Kennedy et al. (*n* = 195) seems to counter the argument that RECIST cannot anticipate survival as it showed responders survived longer in an analysis according to RECIST 1.0: (PR median (95% CI) 25.2 (range, 9.2-49.4) months vs. SD 15.8 (range, 9.3-21.1) months vs. PD 7.1 (range, 6.0-9.5) months (*p* < 0.0001) [40]. In considering response criteria, Chapiro et al. (*n* = 29) then reported that EASL and mRECIST could not reliably predict patient survival (*p* = 0.27 and *p* = 0.44, respectively). Their uni- and multivariate analysis demonstrated that the quantitative European Association for the Study of the Liver (qEASL) response assessment criteria was identified as the sole predictor of patient survival (9.9 months for responders, 6.9 months for non-responders; *p* = 0.038; HR 0.4) [41]. The existing data on which imaging tool is most effective in assessing tumor response to Y-90 treatment has been shown to be controversial, but it is clear that many opportunities exist to establish a standard tool in the field.

**Table 2:** Pre- and Post-Treatment Biomarkers

Pre-Treatment	Post-Treatment
**Imaging**	
*Good outcome*	
cut-off value of 1 for MAA-tumor-to-normal uptake ratio	PET scan response (decreased SUV)
FTV <200 cc	ADC decrease on DWI
TLG <600 g	CT response (decreased diameter, volume, attenuation)
sufficient Y-90 dose to tumor	FTV <30 cc
	TLG <100 g
	**Δ**TLG >26.5
	response via RECIST 1.0
	MRI response
	response via qEASL
**Molecular and Genetic**	
*Good outcome*	
increased albumin	decreased survivin, p53, Bcl-2 expression
*Poor outcome*	
increased HMGB1 levels	increased HMGB1 levels
increased NLR	increased levels of nucleosomes
increased Ang-2 and IL-8	transient increase in VEGF an PDGF-BB
KRAS mut	increased levels of CEA, CA 19-9, CYFRA 21-1, LDH, AST, choline esterase, GGT, alk phos, amylase
increased levels of CEA, CA 19-9, CYFRA 21-1, LDH, AST, choline esterase, GGT, alk phos, amylase	
**Tumor and Patient Characteristics**	
*Good outcome*	
low % liver replacement/ hepatic tumor burden	
low ECOG score	
*Poor outcome*	
extrahepatic disease	
increased bilirubin (>1.3)	
lymphocyte depression	
high LSF	
no liver resection prior to Y90	
summed CT size of largest 2 liver lesions >10 cm	
microscopic lymphovascular invasion of the primary tumor	

A study by Flamen et al. (*n* = 8) prospectively demonstrated that by using a cut-off value of 1 for the MAA-tumor-to-normal uptake ratio, a significant metabolic response could be predicted after Y-90 treatment (sens = 89%, spec = 65%, PPV = 71%, NPV = 87%) [42]. This algorithm is based on the understanding that a relatively cold lesion on MAA-SPECT will be less responsive to radioembolization therapy. Lam et al. (*n* = 25) then demonstrated SPECT-based calculation of dose to tumor (DT) (mean DT, 44.2 Gy) correlated with radiographic response (*p* < 0.001), decrease in serum CEA (*p* < 0.05), and OS (*p* < 0.01). The study showed that patients who received a DT >55 Gy had a median survival of 32.8 mo, compared with 7.2 mo in patients who received less (*p* < 0.05) [43]. Using diffusion weighted imaging to predict therapeutic effects of SIRT, Dudeck et al. (*n* = 21) showed the apparent diffusion coefficient (ADC) decreased significantly in responding lesions by 10.7 +/- 8.4% (*p* < 0.0001) [44]. One other DWI study by Schmeel et al. (*n* = 44) presented data that ADC changes on DWI can predict survival in CRC after SIRT. Other parameters found that were associated with median OS were: optimal functional imaging response (18 vs. 5 mos; *p* < 0.001), hepatic tumor burden <50 % (8 vs. 5 mos; *p* = 0.018), ECOG performance scale <1 (10 vs. 4 mos; *p* = 0.012) and progressive disease according to RECIST (8 vs. 3 mos; *p* = 0.001) [45]. Gulec et al. (*n* = 20), in a prospective trial, demonstrated that pre- and post-treatment functional tumor volume (FTV) and total lesion glycolysis (TLG) were strongly associated with survival. The median survival of the pretreatment cohort with FTV >200 cc was 11.2 months, compared to the cohort with FTV <200cc, whose median survival was 26.9 mos (*p* < 0.05). The median survival of the cohort with pretreatment TLG values >600 g was 11.2 months, as compared to the cohort with TLG <600 g whose median survival was 26.9 mos (*p* < 0.05) [46].

One study by Ulrich et al. (*n* = 66) demonstrated that response to Y-90 radioembolization was found to be independent of the degree of (99m)Tc-MAA uptake and that this should not preclude patients from treatment [47]. Fowler et al. (*n* = 9) looked at the use of PET/MRI dose volume histograms in relation to its ability to predict response. Their data revealed an average dose of 29.8 Gy offered 76.9% sensitivity and 75.9% specificity of tumor response [48]. Barabasch et al. (*n* = 20) compared diffusion-weighted MRI to PET/CT in determining early response to Y-90. The study found that the PPV was 96% for MRI and 88% for PET/CT and the NPV to predict absence was 92% for MRI and 56% for PET. The sensitivity for detecting response was significantly higher for MRI (96%) than for PET (65%) (*p* < 0.02). However, it should be noted that only 20 of the 35 patients studied in this cohort had mCRC [49]. As the field of radiology is inherent to interventional oncology, imaging biomarker studies that consider response and survival are imperatively important to future treatment decisions. Further identification of which imaging modality would best assess treatment response post-Y-90 is still needed.

**Figure 1:**
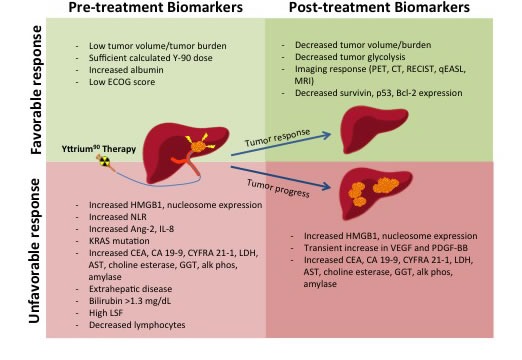
Pre-treatment Biomarkers and Post-treatment Biomarkers

#### Tumor and patient characteristic markers

Some information has also been published on tumor and patient characteristics that can possibly predict tumor response to Y-90 radioembolization treatment in mCRC lesions of the liver. Xing et al. (*n* = 79) recently reported that high lung shunt fraction (LSF) demonstrated significantly poorer survival compared with low LSF in colorectal liver metastases treated with Y-90 (13.5 vs 7.0 months, *p* = 0.013) [50]. This correlation is most likely due to increased vasculature development in advanced tumors and may also be due to decreased dosing as a result of increased LSF on pre-treatment assessments. Deipolyi et al. (*n* = 62) also looked at LSF and determined that patients with higher LSF had significantly decreased survival compared to patients with lower LSF (*p* = .03). This study also showed that patients who received chemotherapy before radioembolization and had low LSF had the longest survival (*p* = .02) [51]. Dunfee et al. (*n* = 130) reported that response seen on imaging 1-month post Y-90 treatment can be a favorable indicator of prognosis. A significant effect on survival was found via multivariate analysis in regards to ECOG performance status >0 (HR = 7.98; 95% CI, 3.98-16), hepatic tumor burden of 51%-75% (HR, 2.46; 95% CI, 1.01-6.02), bilirubin level >1.3 mg/dL (HR, 2.60; 95% CI, 1.27-5.34), response on imaging based on WHO criteria (HR, 0.48; 95% CI, 0.24-0.94), and lymphocyte depression (HR, 0.56; 95% CI, 0.31-0.96) [52]. A retrospective study from Schonewolf et al. (*n* = 30) demonstrated that smaller tumor volumes (<300 mL), were predictive for extrahepatic failure patterns compared with hepatic recurrence (*p* = 0.046) [53]. Tohme et al. (*n* = 107) then looked into age as a possible cause of difference in survival. In that study, no significant difference was found with regard to median OS between younger [8.4 months; 95% confidence interval (CI) = 6.2-10.6] or elderly patients (8.2 months; 95% CI = 5.9-10.5, *p* = 0.667). As expected, presence of extrahepatic disease at time of Y-90 was associated with worse median survival in both age groups [54]. Abbott et al. (*n* = 68) looked into hepatic burden of disease (HBD) and showed median and 2-year OS for patients with ≤25% versus >25% HBD were 19.6 months and 42% versus 3.4 months and 0% (*p* < .0001) [55]. Next, Fendler et al. (*n* = 100) demonstrated that four specific parameters were associated with survival in patients with mCRC receiving Y-90. Reduced patient survival was found via multivariate analysis in the following cohorts: no liver surgery before SIRT (HR:1.81, *p* = 0.014), CEA serum level ≥150 ng/ml (HR:2.08, *p* = 0.001), transaminase toxicity level ≥2.5x upper limit of normal (HR:2.82, *p* = 0.001), and summed computed tomography (CT) size of the largest two liver lesions ≥10 cm (HR:2.31, *p* < 0.001) [56]. Sofocleous et al. (*n* = 53) demonstrated via multivariate analysis that CEA levels greater than, or equal to 90 ng/mL (*p* = 0.004) and microscopic lymphovascular invasion of the primary (*p* = 0.002) are independent predictors of decreased overall survival in patients undergoing Y-90 treatment of CRLM [57]. Understanding which tumor characteristics act as good parameters to use in patient selection is key to better quality patient care, patient outcomes and decreased treatment costs by avoiding unbeneficial procedures.

### Patient selection and future of predictive markers in metastatic CRC

Clinical factors presented in this review have the potential to improve Y-90 patient selection. The most promising of which include known clinical prognostic factors: ECOG status, NLR, Albumin levels, LDH, and tumor burden.

It should be noted that very few “true” biomarkers have been identified to aid in the selection of CRLM patients for Y-90 treatment. True predictive markers such as KRAS and pre-treatment MAA-tumor-to-normal uptake ratio are excellent examples capable of informing Y-90 treatment outcomes. It is important then to identify other accepted CRC biomarkers in the literature to inform prospective research projects. The most commonly used serum biomarker in CRC treatment is CEA. CRC produces larger quantities of the CEA protein and it is thought to be involved in the primary tumor's ability to metastasize. [58] It has been shown to be most effective in assessing for recurrence and response to treatment and has also been shown to be highly sensitive for liver metastases. [58, 59] A number of heritable mutations have been shown to lead to CRC-related polyposis syndromes including mutations in APC, MUTYH, STK11, LKB1, SMAD4, BRMP1A and PTEN genes [60]. Lynch syndrome is the most common heritable form of CRC and has been shown to result from inherited mutations in mismatch repair genes (MMR). The four most commonly mutated MMR genes are MLH1, MSH2, MSH6 and PMS2 [61]. In addition to screening for heritable causes, MMR mutations leading to microsatellite instability have been shown to be a good prognostic indicator with patients having fewer metastases [62]. MMR positive patients have also been shown to respond better to immunotherapy with immune checkpoint inhibitors, presumably due to a higher immunogenicity of these tumors [63]. Research into targeted therapy has shown that specific genetic mutations impact tumor response to individual therapies. CRC tumors with mutations in KRAS and NRAS yield a decreased response to anti-EGFR therapy [64] [65]. Reduced overall survival and progression-free survival have been shown in CRC when the BRAF gene is mutated [66]. Loss of heterozygosity (LOH) and chromosomal instability (CIN), specifically LOH at 18q, have been shown to cause a possible resistance to fluorouracil [67]. Other genes such as PTEN, ERCC1, VEGF, PIK3CA and Top I have been studied but have not shown to be predictive in response to specific therapies or have shown inconsistent research results [68, 69]. A recent meta-analysis by Mei et al. specifically looked at PIK3CA and determined that there is a neutral association with PIK3CA mutation in CRC and patient survival. The data reported showed the summary HRs for OS and PFS were 0.96 (95% CI 0.83–1.12) and 1.20 (95% CI 0.98–1.46), for mut and wt cohorts respectively [70].

These biomarkers have mainly been studied in regards to standard systemic chemotherapy treatments. It is very possible that using these biomarkers, that have been established in a systemic therapy model, could allow for translational research in the field of locoregional therapies such as Y-90 radioembolization, to allow for better optimization of patient selection.

## CONCLUSIONS

There have been strides made in the development of biomarkers that can inform interventionalists on treating mCRC lesions of the liver with Y90 radioembolization. There is still a great need for information, especially genetic tumor markers, that could predict which lesions would be most susceptible to treatment. Additional prospective, large cohort studies on genetic markers such as BRAF, MMR genes and NRAS would greatly benefit the field and help to inform treatment decisions.
